# Genome Sequence of the Basidiomycetous Yeast *Pseudozyma antarctica* T-34, a Producer of the Glycolipid Biosurfactants Mannosylerythritol Lipids

**DOI:** 10.1128/genomeA.00064-13

**Published:** 2013-04-04

**Authors:** Tomotake Morita, Hideaki Koike, Yoshinori Koyama, Hiroko Hagiwara, Emi Ito, Tokuma Fukuoka, Tomohiro Imura, Masayuki Machida, Dai Kitamoto

**Affiliations:** a Research Institute for Innovation in Sustainable Chemistry, National Institute of Advanced Industrial Science and Technology (AIST), Tsukuba, Tsukuba, Ibaraki, Japan; b Bioproduction Research Institute, National Institute of Advanced Industrial Science and Technology (AIST), Tsukuba, Tsukuba, Ibaraki, Japan

## Abstract

The basidiomycetous yeast *Pseudozyma antarctica* T-34 is an excellent producer of mannosylerythritol lipids (MELs), members of the multifunctional extracellular glycolipids, from various feedstocks. Here, the genome sequence of *P. antarctica* T-34 was determined and annotated. Analysis of the sequence might provide insights into the properties of this yeast that make it superior for use in the production of functional glycolipids, leading to the further development of *P. antarctica* for industrial applications.

## GENOME ANNOUNCEMENT

*Pseudozyma antarctica* is an ustilaginomycetous anamorphic basidiomycetous yeast belonging to *Ustilaginomycetes*, which includes the smut fungus *Ustilago maydis* (1, 2). *P. antarctica* T-34 (renamed from *Candida antarctica* T-34) was isolated in Tsukuba, Japan, as a producer of the extracellular glycolipids known as mannosylerythritol lipids (MELs), which consist of 4-O-β-d-mannopyranosyl-*meso*-erythritol as the hydrophilic moiety and fatty acids as the hydrophobic moiety (3). MELs have gained recognition as environmentally friendly biosurfactants, due to their excellent surface activities, and they have also attracted considerable interest in recent years due to their unique properties, including self-assembly, antitumor, and cell differentiation induction activities, as well as their moisturizing and hair-repairing properties (4, 5). Further improvements to the mass production of MELs, and their applications to life sciences, nanotechnology, and environmental technology, are currently being investigated (6–8).

Here, we present the genome sequence of *P. antarctica* T-34 as that of a typical MEL producer. The *P. antarctica* genome was sequenced with 454/Roche sequencing (FLX Titanium) to highly oversample the genome (20-fold coverage), with a total of 1,523,105 reads and the generation of a mate-pair library (insert size of 3 to 4 kb), enabling the assembly of 1,300 contigs into 27 “supercontigs” (scaffolds) using automated shotgun assembly and BLASTn-based contig end joining. The nuclear genome of 18.0 Mb was covered by 27 scaffolds, including 22 scaffolds of >100 kb and 3 scaffolds of >1 Mb.

Protein-coding genes were automatically predicted and the gene models were automatically created for functional annotation, accurate translational start-and-stop assignment, and intron location. This resulted in a set of 6,543 protein-coding genes, of which 4,910 (74.9%) are homologous to sequences in the protein database of the National Center for Biotechnology Information (BLASTp E value, 1e−5; sequence length, ≤20% difference; and sequence identity, ≥50%). A protein function can also be tentatively assigned to about 57.2% of the genes according to the EuKaryotic Orthologous Groups (KOG) classifications. Functions in metabolic pathways were assigned to 4,649 genes using the Kyoto Encyclopedia of Genes and Genomes (KEGG).

The gene cluster responsible for MEL biosynthesis in *P. antarctica* was found on scaffold 19, corresponding to chromosome 7 of *U. maydis* (9). The hydrophobic part, mannosylerythritol, is initially formed by the reaction of an erythritol/mannose transferase gene (*emt1*), and then MELs are produced via the reactions of acyl transferase genes (*mac1* and *mac2*) and an acetyltransferase gene (*mat1*) (10). MELs are possibly secreted by the putative transporter encoded by *mmf1*. The 5 genes of the cluster, *PaEMT1*, *PaMAC1*, *PaMAC2*, *PaMMF1*, and *PaMAT1*, in *P. antarctica* show high levels of identity of 73, 59, 52, 75, and 53% to the corresponding genes in *U. maydis*. These results supports the conclusion that the gene cluster of *P. antarctica* works in the same way as that of *U. maydis*.

### Nucleotide sequence accession numbers.

The nucleotide sequence of the *P. antarctica* genome has been deposited in DDBJ/EMBL/GenBank under the accession no. BAFG01000001 to BAFG01000761 (as 761 entries) and DF196767 to DF196793 (as 27 scaffolds).
